# Neurotoxicity Assessment of Perfluoroundecanoic Acid (PFUnDA) in Developing Zebrafish (*Danio rerio*)

**DOI:** 10.3390/toxics13121012

**Published:** 2025-11-22

**Authors:** Lev Avidan, Cole D. English, Emma Ivantsova, Amany Sultan, Christopher J. Martyniuk

**Affiliations:** 1 Center for Environmental and Human Toxicology, Department of Physiological Sciences, College of Veterinary Medicine, University of Florida, Gainesville, FL 32611, USA; lavidan@ufl.edu (L.A.); coleenglish@ufl.edu (C.D.E.); eivantsova@ufl.edu (E.I.); amanysultan2025@gmail.com (A.S.); 2 Animal Health Research Institute, Agriculture Research Centre, Giza 12619, Egypt; 3 UF Genetics Institute, Interdisciplinary Program in Biomedical Sciences Neuroscience, Gainesville, FL 32611, USA

**Keywords:** zebrafish, neurotoxicity, perfluorinated compounds

## Abstract

Aquatic species are exposed to several long-chain per- and polyfluoroalkyl substances (PFASs) in the environment but their potential toxicity is not well studied. In this study, we assessed the effects of perfluoroundecanoic acid (PFUnDA) exposure on developing zebrafish. To do this, we investigated the potential for oxidative stress and neurotoxicity by measuring reactive oxygen species, apoptosis, gene expression, and locomotor activity. Mortality was evident in fish exposed to 1000 µg/L PFUnDA, and apoptosis was indicated in fish exposed to 100 µg/L PFUnDA via an increase in *casp3* transcription. No change in reactive oxygen species in 7-day-old larval fish exposed to 0.01 up to 1000 µg/L PFUnDA was detected. Visual motor response analysis revealed hypoactivity in different light–dark periods that occurred in a concentration-specific manner. At the transcriptional level, several neurotoxicity-related genes (*casp3*, *bdnf*, *gfap*, *gmfb*, *nkx2-2a*) were significantly upregulated, further supporting neurotoxic effects. Overall, these findings indicate that PFUnDA disrupts neurodevelopment and behavior in zebrafish larvae, raising concerns for this long-chain PFAS.

## 1. Introduction

Chemicals that contain unique properties, such as thermal, oil, and water resistance, are utilized in a variety of applications (i.e., aviation, cookware, electronics). Per- and polyfluoroalkyl substances (PFASs) are chemicals that possess such properties and are characterized as having carbon chains saturated with fluorine atoms. PFASs are environmentally persistent due to relatively long biological half-lives [1] and demonstrate high bioaccumulation potential in organisms. As such, PFASs can have adverse impacts on organisms, including reproductive toxicity [2], endocrine disruption [3], and metabolism [4,5]. PFASs are further categorized by their carbon chain lengths and functional groups. For example, perfluorocarboxylic acids can be further grouped into short- or long-chained compounds, depending on whether the compound contains less than or at least eight carbons, respectively. In general, it has been suggested that increasing carbon chain length is associated with increased toxicity to organisms [6].

Perfluoroundecanoic acid (PFUnDA, C_11_HF_21_O_2_) is an example of a long-chain perfluorocarboxylic acid. Within the past several years, studies have reported on the global occurrence of PFUnDA in environmental matrices. In the United States of America (USA), maximum surface water concentrations of 5.2 ng/L PFUnDA in Las Vegas, Nevada [7] and <1.0 ng/L in New Jersey, USA [8] have been reported. Additionally, in Florida, Camacho et al. [9] report a range of 0.3–114.3 ng/L PFUnDA whereas Griffin et al. [10] report a range of 0.26–0.33 ng/L PFUnDA. Studies of American surface and soil sediment have reported PFUnDA concentrations of 14 µg/kg and 10 µg/kg, respectively [11]. In Asia, PFUnDA concentrations of up to 1.62 ng/L [12] and between 0.05 and 0.26 ng/L [13] were reported in surface and seawater samples, respectively, in China. PFUnDA concentrations of up to 3.52 ng/L were detected in water samples collected from the west coast of Korea [14]. In the Nordic Region, maximum concentrations of 6.5 ng/L, 1.9 ng/L, and <1.2 ng/L PFUnDA were reported in groundwater, river water, and drinking water from a Swedish aquifer, respectively [15]. In Sweden, freshwater samples collected across airports had a maximum concentration of 0.03 ng/L PFUnDA [16] and seepage water samples in Norway were also found to contain 8.6–18 ng/L PFUnDA [17]. Within aquatic sources at locations near American air force bases, PFUnDA was observed at a concentration of 0.086 µg/L in the groundwater and 0.21 µg/L in the surface water [11]. Taken together, this PFAS has been identified in water systems on a global scale.

Some studies also report the presence of PFUnDA in sediment at concentrations of up to 3.8 ng/g [18] and 22.9 ng/g [7] in Georgia and Nevada, USA, respectively. Additionally, PFUnDA ranged from 0.10 to 2.14 ng/g in sediment collected from New Jersey, USA [8], whereas a lower range of 0.02–0.43 ng/g was reported in Florida sediment [10]. In the St. Lawrence River, Quebec, Canada, PFUnDA was found at concentrations of up to 0.071 ng/g dw [19], and PFUnDA concentrations of up to 4.5 ng/g dw were reported from a Swedish aquifer [15]. In China, PFUnDA ranged from 0.03 to 0.13 ng/g dw in the Yangtze River Estuary, China [13], and, from the Port of Santos, Brazil, 138 pg/g dw of PFUnDA was detected [20]. Thus, both water and sediment can contain detectable levels of PFuNDA.

Studies have also evaluated the presence of PFUnDA within fish tissues. In filets of bluegill sunfish (*Lepomis macrochirus*) collected from rivers in Minnesota and North Carolina, USA [21], the wet weight of PFUnDA ranged from 0.53–6.02 ng/g to 1.31–50.5 ng/g, respectively. Other fish species collected from rivers in New Jersey had mean concentrations of between 0.5 and 8 ng/g PFUnDA [8], and maximum concentrations of 45.6 ng/g and 18 ng/g PFUnDA have been reported in fish collected from USA urban river and Great Lakes, respectively [22]. From South Carolina, USA, spot (*Leiostomus xanthurus*), red drum (*Sciaenops ocellatus*), Atlantic croaker (*Micropogonias undulatus*), striped mullet (*Mugil cephalus*), and spotted seatrout (*Cynoscion nebulosus*) were analyzed: the average relative percentage of PFUnDA within whole and muscle filet samples ranged from 4.97–21.7 to 1.39–8.53, respectively [23]. In fish collected from the St. Lawrence River, Quebec, Canada, including species such as bluntnose minnow (*Pimephales notatus*), smallmouth bass (*Micropterus dolomieu*), and yellow perch (*Perca flavescens*), there was a 100% detection frequency of PFUnDA and it ranged from 0.45 to 6.8 ng/g ww [19]. Lastly, 140 ng/g fresh weight PFUnDA was detected in trout liver from Norway [17], and up to 19.2 ng/g wet weight PFUnDA was detected in fish from Beijing, China [24]. Overall, PFUnDA’s widespread detection across multiple fish species and regions highlights its persistence, potential for bioaccumulation, and relevance as a concerning environmental contaminant.

To date, limited studies have exposed aquatic organisms to PFUnDA, and, to our knowledge, this is the second study to expose developing zebrafish to PFUnDA. Zebrafish are used as experimental models for several purposes, one of which is that they allow large-scale experimental analysis due to their high fecundity and easy handling and another reason is that they contain the same neurotransmitters and a central nervous system (CNS) structure as humans [25]. Thus, to characterize the potential sublethal toxicity of PFUnDA, we completed a continuous 7-day PFUnDA exposure to developing zebrafish embryos and larvae and measured reactive oxygen species (ROS) production, apoptosis, gene expression, and locomotor alterations.

## 2. Materials and Methods

### 2.1. Chemical Preparation

PFUnDA was purchased from Millipore Sigma (CAS no. 2058-94-8, Pharmaceutical Secondary Standard; Certified Reference Material; St. Louis, MO, USA). Stock solutions were prepared in 0.1% dimethyl sulfoxide (DMSO) (CAS: 67-68-5, purity ≥ 99.9%, Sigma-Aldrich, St. Louis, MO, USA) and added to embryo rearing media (ERM) containing the zebrafish embryos. ERM was prepared as described in Westerfield [26]. Exposure solutions were prepared daily to yield final nominal concentrations of 0.01, 1, 10, 100, and 1000 μg/L PFUnDA (final of <0.1% *v*/*v* DMSO in treatments).

### 2.2. Husbandry and Egg Production of Zebrafish

Adult zebrafish (AB x Tübingen, *Danio rerio*, 6 months of age) were raised in a flow-through Pentair system at the University of Florida. Environmental conditions for zebrafish breeding have been outlined previously [27,28]. Staging of embryos followed that of Kimmel, Ballard [29]. Institutional Animal Care and Use Committee of University of Florida approved all experiments (UF IACUC#201708562). Details on breeding and husbandry conditions are provided in Supplemental Methods.

### 2.3. PFUnDA Exposure Design

Zebrafish acute toxicity tests with embryos and larval fish followed guidelines by the Organization for Economic Co-operation and Development (OECD) [30] with modification. For each independent experiment (6 experiments), 15–20 normally developing embryos were randomly assigned to 25 mL Pyrex glass beakers. An amount of 11 mL of sterile ERM was added at a final concentration of 0.01, 1, 10, 100, or 1000 µg/L PFUnDA. A concentration of 0.01 µg/L can be considered environmentally relevant; however, our tested concentrations of 0.01 µg/L up to 1000 µg/L allowed for a broad range of possible sublethal effects due to PFUnDA, and we aimed to determine if there was acute toxicity at 1000 µg/L. Imaging and endpoint description is provided in Supplemental Methods. Experiments were replicated to obtain enough fish to conduct all toxicity assays and sublethal endpoints. Tricaine mesylate (Syncaine, Tricaine-S, Pentair Aquatic Eco-Systems, Inc., Apopka, FL USA) was used for euthanasia at 250 mg/L buffered with equal-part sodium bicarbonate to a pH of between 7.0 and 7.5.

### 2.4. Acridine Orange

The acridine orange (AO) staining method was conducted to detect apoptotic cells in zebrafish larvae (*n* = 14–16/treatment) and is described in the Supplemental Methods.

### 2.5. Reactive Oxygen Species

Embryos were obtained for ROS assessment as outlined above in Section 2.2. The methods were performed following our protocol, which is provided in the Supplemental Methods. Treatment groups included 0.1% DMSO, ERM, 0.01, 1, 10, 100, and 1000 µg/L PFUnDA (μg/mL media/protein) (*n* = 3 to 5 beakers per experimental group).

### 2.6. Real-Time PCR Analysis

A double batch of fish were bred as per Section 2.2 for real-time PCR. Details on breeding are included in the Supplemental Methods. Real-time PCR analysis included 0.1% DMSO, 1 µg/L, and 100 µg/L PFUnDA; *n* = 3 or 4 biological replicates per group. Biological replicates varied due to balancing replicates (beakers), concentrations, and larval fish numbers. Only two tested concentrations were investigated (1 µg/L and 100 µg/L). We were limited in the number of fish; therefore, we aimed to keep the number of larval fish high in beakers to obtain more RNA but did not have enough larval fish to do all the concentrations. Real-time PCR was performed following our established protocols using TRIzol^®^ Reagent (Life Technologies, Carlsbad, CA, USA) [31]. Samples (7-day old larval fish pool from each experimental beaker) were run in duplicate and followed RT-qPCR cycling parameters described by us [32].

Primer sequences were obtained from the published literature (Supplemental Table S1) [32,33,34,35,36,37,38,39,40,41,42,43,44,45,46,47]. Transcripts measured included *ache* (acetylcholinesterase), *atp06* (ATP synthase F0 subunit 6), *bcl2* (B-cell lymphoma 2), *bdnf* (brain-derived neurotrophic factor), *casp3* (caspase 3), *cat* (catalase), *ctgfa* (connective tissue growth factor a), *elavl3* (ELAV-like RNA binding protein 3), *gap43* (growth-associated protein 43), *gfap* (glial fibrillary acidic protein), *gmfb* (glia maturation factor beta), *ho1* (heme oxygenase 1), *keap1* (Kelch-like ECH-associated protein 1), *manf* (mesencephalic astrocyte-derived neurotrophic factor), *mbp* (myelin basic protein), *nestin* (intermediate filament protein nestin), *nkx2-2a* (NK2 homeobox 2a), *nqo1* (NAD(P)H quinone dehydrogenase 1), *nrf2* (nuclear factor erythroid 2-related factor 2), *sod1* (superoxide dismutase 1), *sod2* (superoxide dismutase 2), *sox 19b* (SRY-box transcription factor 19b), and *tubulin* (tubulin). *Rps18* (ribosomal subunit 18) and *bactin* (beta-actin) were used to normalize expression levels of all target genes using the CFX Manager (v3.1) software. Normalized expression was obtained for each target gene using CFX Manager™ software (v3.1) (baseline subtracted), and the cycle threshold (Cq) method was employed.

### 2.7. Visual Motor Response Test

The VMR test proceeded as per our established methods [27,31,48,49]. In each trial, ~800 zebrafish embryos at 6 hpf were randomly assigned to an experimental group of either 0.1% DMSO, ERM control, or 0.1–1000 μg/L PFUnDA (*n* = 120–200 fish derived from replicate beakers/treatment, in 3 independent experiments). An 80% water change was conducted daily to renew the PFUnDA. The experiments were conducted at a temperature of 27 ± 1 °C and photoperiod of 14:10 h in benchmark mini ovens. For each trial, the 0.1% DMSO group was adjusted to become a relative measure of 1 (by dividing individual values by the mean of the group), and all other experimental groups were adjusted to be relative the DMSO control (by dividing data for each fish by the mean DMSO control value). In this manner, the three experiments could be combined into a single graph.

### 2.8. Statistical Analysis

Statistical analysis and graphing were conducted using GraphPad v9.5.1 (La Jolla, CA, USA). Data were first assessed for normality using a Shapiro–Wilk test, and ROS, gene expression data, and “distance moved” (locomotor activity) were log10 transformed to approximate a normal distribution. Survival was analyzed with a Kaplan–Meier test (log-rank Mantel–Cox test). Deformity and hatch data were observably unaltered at the concentrations tested; deformity frequency was low (<2%) in all treatment groups. Apoptosis (AO stain), ROS [relative fluorescence units (µg/mL protein)], gene expression levels, and the VMR for larval zebrafish were analyzed using a One-Way ANOVA (Dunnett’s multiple comparisons test) to the DMSO control (mean ± SD). Significance of difference was determined to be *p* < 0.05.

## 3. Results

### 3.1. Survival and Deformity

**Figure 1:**
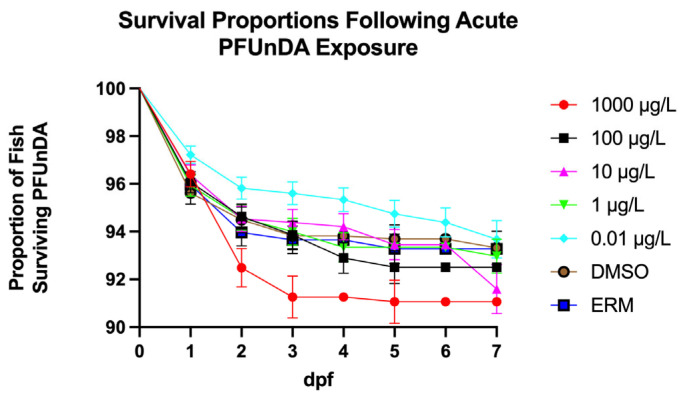
Mean number of embryo/larvae surviving PFUnDA over a 7-day exposure period (Log-rank (Mantel–Cox) test, *n* = 2409 individual fish, and data were combined over three experiments). The error bars on the graph represent the S.E.M. Errors bars are within circles for some points on the graph.

**Figure 2:**
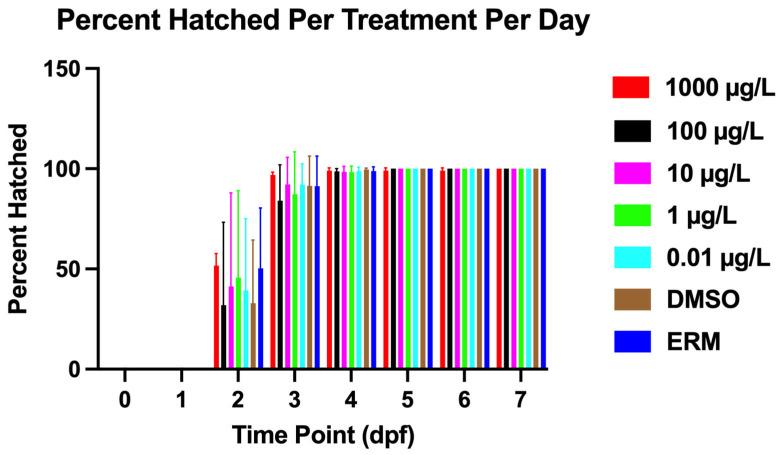
Percent of hatched embryos over a 7-day exposure period following PFUnDA exposure (Log-rank (Mantel–Cox) test, *n* = 2409 individual fish, and data were combined over three experiments). The error bars on the graph represent the S.D.

**Figure 3:**
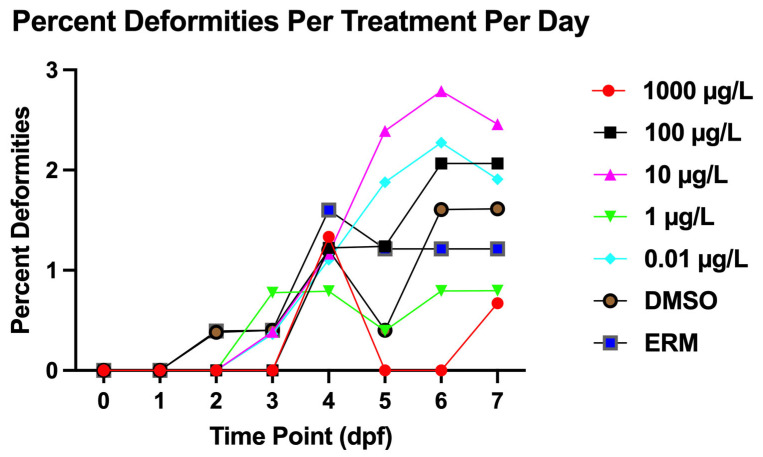
Percent of deformities observed in zebrafish embryos/larvae exposed over a 7-day exposure period to PFUnDA (*n* = 2409 individual fish, and data were combined over three experiments).

Zebrafish larvae exhibited notable mortality compared to controls, with a Log-rank (Mantel–Cox) test revealing the most significant decrease in survival in the 1000 µg/L concentration (Chi-square = 13.37, d.f. = 6, *p* value = 0.0375). However, overall survival was relatively comparable to DMSO controls (Figure 1). Mortality was low and survival was <90% for all treatments. Thus, survival of embryos or larvae were not affected by <1000 µg/L. PFUnDA did not affect hatch rates (Figure 2). Deformity frequency in controls was <0.655%, marked by caudal tail malformations, pericardial edema, lordosis, kyphosis, and yolk sac edema (Figure 3). Treatments did not differ in deformity frequency. Axial malformations (lordosis and kyphosis) were observed in some cases as fish swam within the beaker.

### 3.2. Acridine Orange

**Figure 4:**
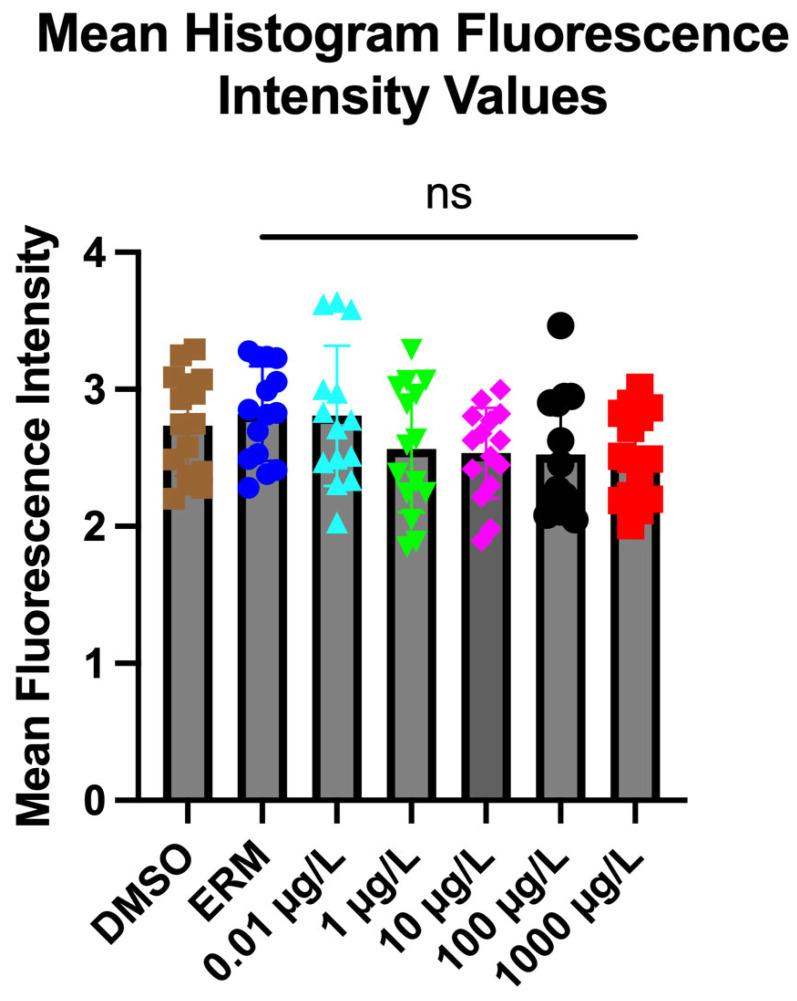
Mean fluorescence intensity in zebrafish larvae exposed to 0.1% DMSO, ERM, 0.01, 1, 10, 100, and 1000 µg/L PFUnDA at 7 dpf. Data represented as the mean value (±SD) (one-way ANOVA followed by Dunnett’s post hoc test, *n* = 14–16/treatment).

There were no significant alterations noted following exposure to any concentration of PFUnDA versus the DMSO control group (One-way ANOVA, Dunnett’s post hoc test) (F _(6, 97)_ = 1.764, *p* = 0.1146) (Figure 4).

### 3.3. Reactive Oxygen Species

**Figure 5:**
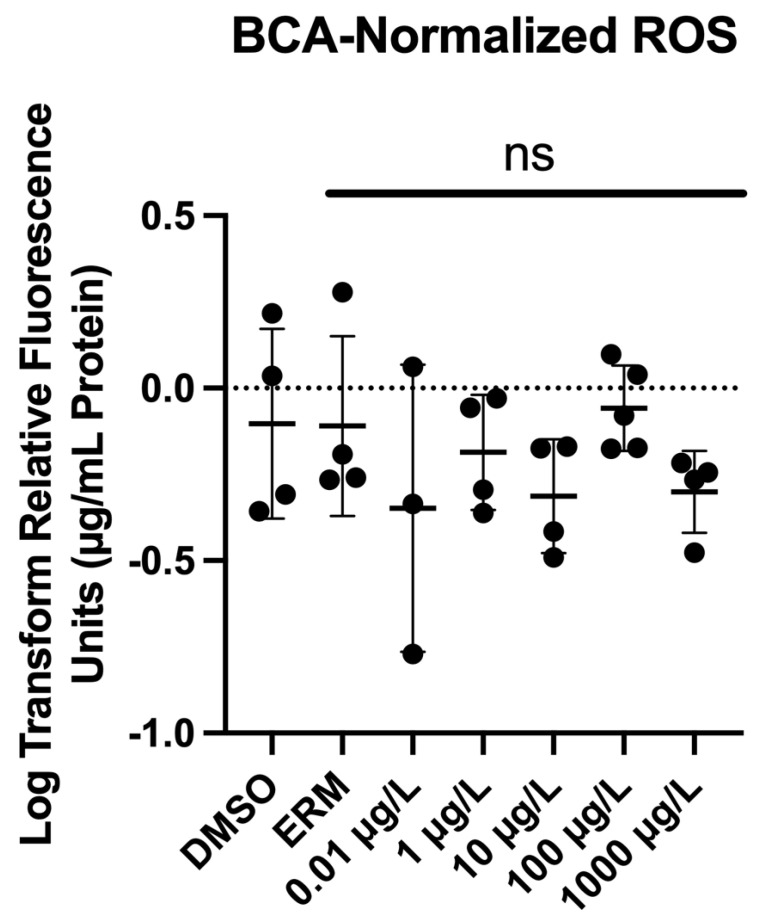
Normalized reactive oxygen species (ROS) following exposure to 0.1% DMSO, ERM, 0.01, 1, 10, 100, and 1000 µg/L PFUnDA (μg/mL media/protein). Each circle represents a biological replicate; the mean (±S.D.) is represented by the horizontal line (One-Way ANOVA, Dunnett’s multiple comparisons test, *n* = 3–5/experimental group).

Zebrafish larvae were exposed to 0.01, 1, 10, 100, or 1000 µg/L PFUnDA for 7 days. ROS levels did not differ in 7 dpf larvae relative to the 0.1% DMSO control (F _(6, 21)_ = 1.113, *p* = 0.3882) (Figure 5).

### 3.4. Real-Time PCR Analysis

**Figure 6:**
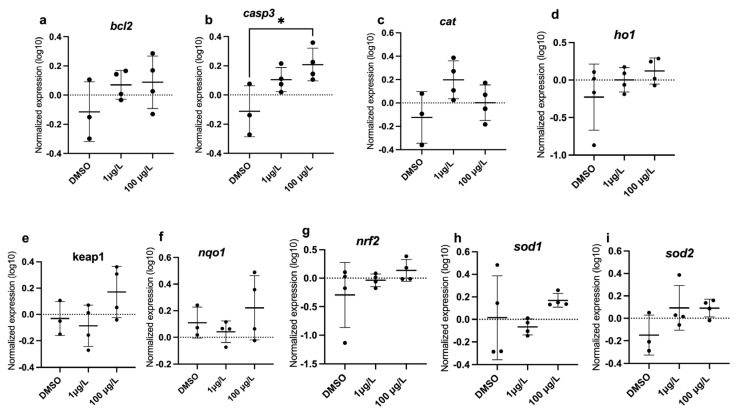
Relative oxidative stress gene expression in zebrafish larvae exposed to solvent control (0.1% DMSO), 1 µg/L, or 100 µg/L PFUnDA. (**a**) *bcl2*, (**b**) *casp3*, (**c**) *cat*, (**d**) *ho1*, (**e**) *keap1*, (**f**) *nqo1*, (**g**) *nrf2*, (**h**) *sod1*, and (**i**) *sod2*. Data are represented as mean ± standard deviation. Asterisks denote significant difference compared to the DMSO control within each interval (* *p* < 0.05) (One-Way ANOVA, Dunnett’s multiple comparisons test, *n* = 3–4 per treatment).

**Figure 7:**
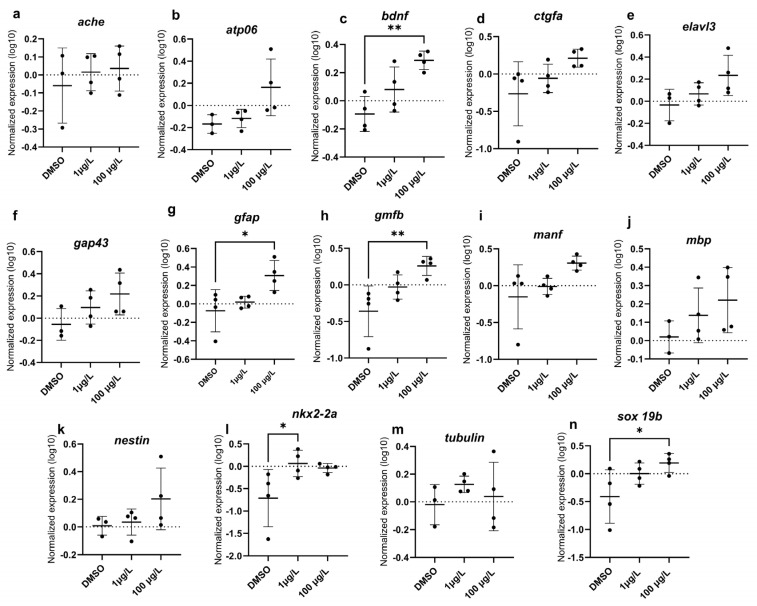
Relative expression of neurotoxic genes in zebrafish larvae exposed to either the solvent control (0.1% DMSO), 1 µg/L, or 100 µg/L PFUnDA. (**a**) *ache*, (**b**) *atp06*, (**c**) *bdnf*, (**d**) *ctgfa*, (**e**) *elavl3*, (**f**) *gap43*, (**g**) *gfap*, (**h**) *gmfb*, (**i**) *manf*, (**j**) *mbp*, (**k**) *nestin*, (**l**) *nkx2-2a*, (**m**) *tubulin* (*tubb3*), (**n**) *sox 19b*. Data are represented as mean ± standard deviation. Asterisks denote a significant difference compared to the DMSO control within each interval (* *p* < 0.05, ** *p* < 0.01) (One-Way ANOVA, Dunnett’s multiple comparisons test, *n* = 3–4 per treatment).

**Figure 8:**
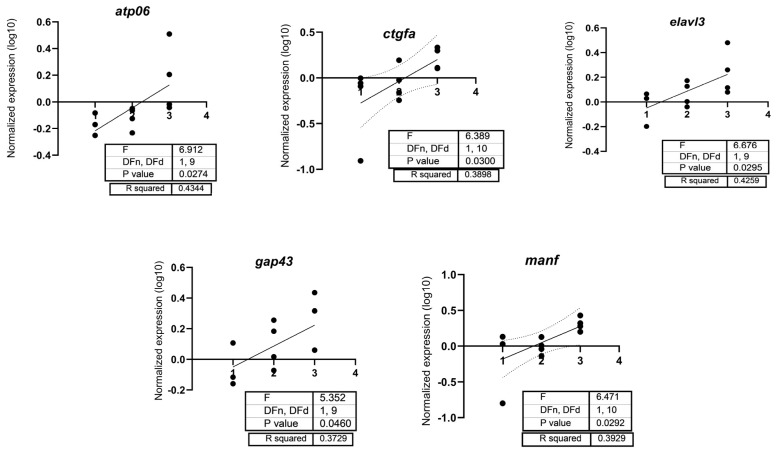
Linear regression analysis of *atp06*, *ctgfa*, *elavl3*, *gap43*, and *manf* in 7-day old larval zebrafish exposed to either solvent control (#1 on x-axis = 0.1% DMSO), #2 on x-axis = 1 µg/L, or #3 on x-axis = 100 µg/L PFUnDA. Each circle represents a biological replicate.

Oxidative stress (*bcl2*, *casp3*, *cat*, *ho1*, *keap*, *nqo1*, *nrf2*, *sod1*, *sod2*) genes were measured, and only *casp3* was significantly upregulated with 100 µg/L PFUnDA (F_(2, 9)_= 5.942, *p* = 0.0262) (Figure 6). Regarding transcripts related to neurotoxicity, *bdnf* (F_(2, 9)_= 9.622, *p* = 0.0058), *gfap* (F_(2, 9)_= 5.757, *p* = 0.0245), *gmfb* (F_(2, 9)_= 7.002, *p* = 0.0147), and *sox19b* (F_(2, 9)_= 3.857, *p* = 0.061) were upregulated with 100 µg/L PFUnDA, while 1 µg/L PFUnDA increased expression levels of *nkx2.2a* (F_(2, 9)_= 3.795, *p* = 0.0638) (Figure 7). It is important to note that following a post hoc Dunnett’s test, the expression of both *sox19b* and *nkx2.2a* were induced. There was no change in *ache*, *atp06*, *ctgfa*, *elavl3*, *gap43*, *manf*, *mbp*, *nestin*, and *tubulin*). Regression analysis revealed increased expression of *atp06*, *ctgfa*, *elavl3*, *gap43*, and *manf* (Figure 8). Taken together, several key transcripts in neuronal differentiation and function were altered by PFUnDA exposure.

### 3.5. Locomotion Activity with Visual Motor Response Test

**Figure 9:**
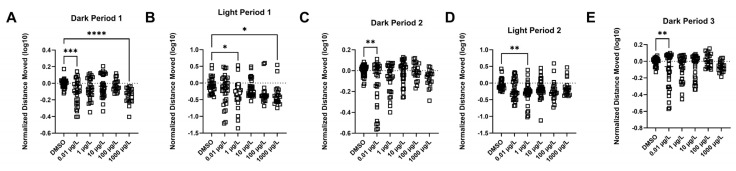
Visual motor response test (VMR) results. Light–dark cycles are organized in 10 min intervals. (**A**) Dark Period 1, (**B**) Light Period 1, (**C**) Dark Period 2, (**D**) Light Period 2, (**E**) Dark Period 3. Mean (±SD) distance moved (mm) per minute interval per experimental group (on a log10 scale). Asterisks denote significant difference compared to the 0.1% DMSO control within each interval (* *p* < 0.05, ** *p* < 0.01, *** *p* <0.001, **** *p* <0.0001) (One-Way ANOVA, Dunnett’s multiple comparisons test, *n* = 16 fish per each of the VMR assays, for a total of 48 fish).

Three different batches of fish were used, and the data were combined into a single graph using a “normalized distance moved” relative to the 0.1% DMSO control group in each time interval. Nominal concentrations of 0.01 up to 1000 µg/L of PFUnDA were tested (Figure 9). In the first dark period (F _(5, 154)_ = 6.321, *p* = <0.0001), locomotor activity was decreased in fish exposed to 0.01 and 1000 µg/L PFUnDA. The second dark period (F _(5, 154)_ = 3.263, *p* = 0.008) and the third dark period (F _(5, 154)_ = 3.441, *p* = 0.0056) featured decreased locomotor activity for larval fish exposed to 0.01 µg/L PFUnDA. During the first light period (F _(5, 154)_ = 3.112, *p* = 0.0105), a decrease in locomotor activity in fish exposed to 1 and 1000 µg/L PFUnDA was observed. In the second light period, (F _(5, 154)_ = 3.103, *p* = 0.0107), there was a decrease in locomotor activity in the 1 µg/L PFUnDA treatment. Taken together, there were concentration- and time-dependent alterations in locomotor activity, with a decrease across concentrations of 0.01 µg/L (in dark periods 1, 2, and 3), 1 µg/L (in light periods 1 and 2), and 1000 µg/L (in dark period 1 and light period 2).

## 4. Discussion

The sublethal effects of perfluoroundecanoic acid (PFUnDA) on developing zebrafish was investigated in the current study to address knowledge gaps regarding its acute toxicity. We observed that PFUnDA at the highest concentration tested (1 mg/L) slightly decreased survival rate. With respect to deformity rates, no significant differences were observed among tested concentrations. However, the lowest percentage of deformities (<0.543%) were observed following exposure to 1000 µg/L PFUnDA (the concentration with the lowest survival rate). As such, this deformity data may indicate a selection effect, in which zebrafish embryos that were severely deformed died earlier in the exposure. Few studies evaluate the impact of PFUnDA exposure on fish. Kim et al. [50] found that eyeball size was significantly decreased, swim bladders were deflated, and yolk sac size was increased in zebrafish exposed to 0.03, 0.1, or 0.3 mg/L PFUnDA for 5 days; survival, hatch rate, and hatch percentage were not significantly impacted. Additionally, Gui et al. [51] exposed zebrafish embryos to various PFASs in which embryos were exposed to up to 10 mg/L PFUnDA until 120 hpf. PFUnDA exhibited the highest toxicity among the PFASs examined in their study, with an EC50 of 4.36 mg/L, which was higher than the concentrations tested in the current study. In the study by Gui and colleagues, pericardial and yolk sac edema, spinal curvature, and uninflated swim bladders, as well as reduced hatchability, were observed. Other studies report similar alterations in other long-chain PFASs close in length to PFUnDA, including perfluorodecanoic acid (PFDA, C_10_HF_19_O_2_) and perfluorononanoic acid (PFNA, C_9_HF_17_O_2_), in fish species, but more studies evaluating PFNA are available. For instance, embryonic zebrafish exposed to 2.0 µM (928.1 µg/L) PFNA until 5 dpf significantly reduced body length and increased yolk sac size, but no abnormalities were observed at the lower concentrations tested (0.02 and 0.2 µM or 9.28 and 92.8 µg/L) [52]. In another study, hatching rate and ventricular edema decreased and increased, respectively, in a dose-dependent manner in zebrafish embryos exposed to 25–400 µM (11.6–185.6 mg/L) PFNA for 96 h [53]. Taken together, PFUnDA does not appear to induce mortality at concentrations below the mg/L concentration, but it can negatively impact other apical endpoints.

We found that 0.01–1000 µg/L PFUnDA did not induce apoptosis in zebrafish. No other studies, thus far, have measured apoptosis in fish following PFUnDA exposure. In a recent study completed by Qian et al. [54] on zebrafish embryos, 100 μM (46.4 mg/L) PFNA was found to induce apoptosis through lipid accumulation. Further studies examining metabolic dysfunction following PFUnDA are thus warranted.

ROS production is indicative of oxidative stress. To our knowledge, this is the first study to measure ROS levels in zebrafish following PFUnDA exposure. We observed no change in ROS levels in zebrafish exposed to a range of PFUnDA concentrations; however, other long-chain PFASs like PFNA (nine carbons) have been noted to induce oxidative stress. For example, zebrafish embryos exposed to 25 to 400 µM (11.6–185.6 mg/L) PFNA for 96 h [53] and to 0.1, 1, or 10 µg/L of PFNA for 5 days [55] were all found to exhibit increased ROS production. Other indicators of oxidative stress, including superoxide dismutase and malondialdehyde, have been reported to be increased following PFNA exposure in zebrafish [55,56,57]. Both PFUnDA and PFNA are long-chain PFAS chemicals, but they do differ by two carbons. This could be the reason for the discrepancy in ROS induction. The studies with PFNA also exposed fish to very high levels (>10 mg/L), and in our study, the highest concentration tested was 1 mg/L. Thus, ROS induction may require higher levels of PFUnDA.

Long-chain PFASs have been noted to disrupt the expression of genes related to neurotoxicity [58,59], oxidative stress [58,60,61], apoptosis [58,62], and the immune system [61,63]. We evaluated apoptosis, oxidative stress, and neurotoxicity-related transcripts following PFUnDA exposure and found only one transcript related to apoptosis that was significantly upregulated (*casp3*), while several neurotoxicity-related transcripts (*bdnf*, *gfap*, *gmfb*, and *nkx2-2a*) were upregulated at select PFUnDA concentrations, indicating transcriptional evidence of neurotoxicity. *Casp3*, a cysteine-aspartic acid protease, is a pro-apoptotic signal that binds caspase 8 and 9 [64], and studies show that it increases with exposure to PFASs [65,66]. In our study, casp3 transcription increased with 100 µg/L PFUnDA, while our AO assay did not detect increased apoptosis. This may suggest an early molecular response in which gene activation occurred prior to visible signs of apoptosis. Alternatively, this difference could be due to the timing of sample collection as AO staining may only detect apoptosis during its peak activity. *Bdnf* is a neurotrophin that contributes towards neuronal survival, differentiation, and synaptic plasticity. It plays an essential role in learning and memory by modulating synaptic strength and long-term potentiation. Its dysregulation has been linked to neurodegenerative diseases and mood disorders, making it a key biomarker of neural health [67]. *Gfap* is used as a glial activation marker as it is an intermediate filament protein expressed in astrocytes. It maintains the structural integrity of astrocytes and supports neuron function, with increased expression marking neuroinflammation following CNS injury [68]. *Gmfb* is a growth and differentiation factor predominantly expressed in the central nervous system, where it regulates glial cell maturation. It has been implicated in neuroinflammatory responses, particularly in the activation of astrocytes and microglia. Overexpression of *gmfb* has been associated with oxidative stress and neurodegenerative conditions [69]. Lastly, *nkx2-2a* is critical for oligodendrocyte differentiation and myelination in the CNS, as it defines ventral neural progenitor identity during embryonic development. Disruption to its expression can thus impair oligodendrocyte lineage commitment and neurological development [70]. Overall, transcript data surrounding PFUnDA are lacking, but one study found that PFUnDA disrupted thyroid-regulating genes, including uridine diphosphate glucuronosyltransferase (*ugt1ab*) upregulation, which suggests accelerated metabolic elimination of thyroid hormone [50].

In our study, behavioral impacts were observed, with concentration-dependent hypoactivity across light and dark phases where effects were most consistent at 0.01 µg/L PFUnDA, with additional hypoactivity at 1 µg/L and 1000 µg/L PFUnDA. Various studies report locomotor alterations in zebrafish exposed to many different PFASs [71,72,73]; however, locomotor data regarding PFUnDA remains limited. One study completed photomotor response at 24 hpf and 120 hpf, respectively, in zebrafish exposed to a variety of PFASs [71]. PFUnDA did not elicit any behavioral alterations in fish exposed to a single concentration of 0.44 µM (248.2 µg/L), but perfluorobutanoic acid (PFBA), perfluorododecanoic acid (PFDoA), PFNA, perfluorooctane sulfonic acid (PFOS), and perfluorotetradecanoic acid (PFTeDA) were found to induce either hyperactivity or hypoactivity at similar concentrations.

## 5. Conclusions

In conclusion, PFUnDA is a long-chain perfluorocarboxylic acid that has been detected worldwide due to its wide-ranging industrial uses. In the current study, PFUnDA exposure was found to have no impact on apoptosis and ROS levels as well as combined hatch and deformity rates in larval zebrafish. However, PFUnDA was observed to slightly decrease survival at the highest concentration tested (1000 µg/L). PFUnDA was also observed to induce hypoactivity as well as upregulate gene transcripts relating to apoptotic and neurotoxic endpoints. There is a possibility that long-term PFUnDA exposure to zebrafish may cause more severe, cumulative effects than short-term exposure, as long-chain PFASs generally have longer half-lives and greater bioaccumulation potential. To date, very few studies have investigated the effects of PFUnDA in aquatic organisms, and, to our knowledge, our work represents only the second study to expose developing zebrafish to PFUnDA. As long-term exposure data for PFUnDA are lacking, our short-term exposure study provides a foundation for understanding PFUnDA’s potential biological impacts.

Overall, these findings indicate that PFUnDA disrupts neurodevelopment and behavior in zebrafish larvae, raising concerns for this long-chain PFAS. One limitation of our study is that nominal concentration was reported and PFUnDA was not directly measured in the exposure water across the range of concentrations tested. For example, PFASs may adsorb to glassware, and they can also sorb to organic matter present in the beaker. To maintain constant exposure, ERM containing PFUnDA was renewed every day from the same stock vial at 90% water change to maintain concentration. In addition, chemical analysis would validate the exposure concentrations of PFUnDA. Another interesting finding was the non-monotonic responses observed in both the locomotor activity assays and several gene expression endpoints. These types of responses may be due to differences in uptake kinetics for PFUnDA in the zebrafish larvae or concentration-dependent effects on receptors or proteins (positive and/or negative feedback). Nevertheless, these measured sublethal responses may be useful to guide the design of future chronic and/or multigenerational studies for environmental risk assessment.

## Data Availability

The original contributions presented in this study are included in the article/Supplementary material. Further inquiries can be directed to the corresponding author.

## Supplementary Materials

The following supporting information can be downloaded at https://www.mdpi.com/article/10.3390/toxics13121012/s1, Table S1: Primers used for real-time PCR analysis. Supplemental Methods: Detailed description of all methods [26,27,28,29,30,31,32,33,34,35,36,37,38,39,40,41,42,43,44,45,46,47,48].

## Abbreviations

ANOVA	Analysis of variance
AO	Acridine orange
BCA	Bicinchoninic acid
CDNA	Complementary deoxyribonucleic acid
CNS	Central nervous system
Cq	Cycle Threshold
DMSO	Dimethyl sulfoxide
DPF	Days post fertilization
ERM	Embryo rearing media
GFP	Green fluorescent protein
HPF	Hours post fertilization
H2-DCFDA	2′,7′-Dichlorodihydrofluorescein diacetate
IACUC	Institutional Animal Care and Use Committee
NRT	No reverse transcriptase
OECD	Organization for Economic Co-operation and Development
PBS	Phosphate-buffered saline
PCR	Polymerase chain reaction
PFASs	Per- and polyfluoroalkyl substances
PFBA	Perfluorobutanoic acid
PFDoA	Perfluorododecanoic acid
PFNA	Perfluorononanoic acid
PFOS	Perfluorooctane sulfonic acid
PFTeDA	Perfluorotetradecanoic acid
PFUnDA	Perfluoroundecanoic acid
RNA	Ribonucleic acid
ROS	Reactive oxygen species
RT-qPCR	Reverse transcription quantitative polymerase chain reaction
SD	Standard deviation
USA	United States of America
VMR	Visual Motor Response
ZIRC	Zebrafish International Resource Center
