# Supplementary material for: Neurotoxicity Assessment of Perfluoroundecanoic Acid (PFUnDA) in Developing Zebrafish (Danio rerio)
Source: Toxics. 2025 Nov 22;13(12):1012. doi: 10.3390/toxics13121012 (PMC12737132; doi:10.3390/toxics13121012)
Supplement: Supplementary file 1 [file toxics-13-01012-s001.zip › Suppl. Methods.pdf]

toxics

MDPI

Article

Neurotoxicity Assessment of Perfluoroundecanoic Acid (PFUnDA) in Developing Zebrafish (*Danio rerio*)

Lev Avidan [1,†], Cole D. English [1,†], Emma Ivantsova [1], Amany Sultan [1,2] and Christopher J. Martyniuk [1,3,*]

[1] Center for Environmental and Human Toxicology, Department of Physiological Sciences, College of Veterinary Medicine, University of Florida, Gainesville, FL 32611, USA; lavidan@ufl.edu (L.A.); coleenglish@ufl.edu (C.D.E.); eivantsova@ufl.edu (E.I.); amanysultan2025@gmail.com (A.S.)
[2] Animal Health Research Institute, Agriculture Research Centre, Giza 12619, Egypt
[3] UF Genetics Institute, Interdisciplinary Program in Biomedical Sciences Neuroscience, Gainesville, FL 32611, USA
* Correspondence: cmartyn@ufl.edu
† These authors contributed equally to this work.

2. Materials and Methods

2.1. Chemical Preparation

PFUnDA was purchased from Millipore Sigma (CAS no. 2058-94-8, Pharmaceutical Secondary Standard; Certified Reference Material). Stock solutions were prepared in 0.1% dimethyl sulfoxide (DMSO) (CAS: 67-68-5, purity ≥99.9 %, Sigma-Aldrich, St. Louis, MO, USA) and added to embryo rearing media (ERM) containing the zebrafish embryos. ERM was prepared as described in Westerfield [1]. Exposure solutions were prepared daily to yield final nominal environmentally relevant concentrations of 0.01, 1, 10, 100, and 1000 µg/L PFUnDA with a final concentration <0.1% *v*/*v* DMSO in experimental treatments.

2.2. Husbandry and egg production of zebrafish

Adult zebrafish (AB x Tübingen, *Danio rerio,* 6 months of age) were raised in a flow-through Pentair system in the Cancer-Genetics Research Center at the University of Florida and are derived from an ongoing zebrafish breeding colony. The colony is infused with new breeders from Zebrafish International Resource Center (ZIRC) every 3 to 4 generations to maintain genetic variation. Environmental conditions for zebrafish breeding have been previously described [2, 3]. Staging of embryos followed that of Kimmel, Ballard [4]. Fish are maintained at a temperature of 27±1 °C, air saturation at 82%, water pH between 7.2 and 7.3, conductivity of 1,500–1,600 µS/cm, saturated oxygen concentration of ~85% of air saturation, and a 14:10 h light/dark cycle. Institutional Animal Care and Use Committee of University of Florida approved all experiments (UF IACUC#201708562). To produce embryos for this study, two females and two males were placed into a standard zebrafish breeding tank with a divider. The following morning at approximately 8 a.m., the divider was removed, and the fish were allowed to breed. Embryos were collected and maintained in the filtered system water, and multiple water changes were conducted to wash the embryos to lower the risk of contamination.

2.3. PFUnDA exposure regime

The embryonic acute toxicity test followed recommendations from the Organisation for Economic Co-operation and Development (OECD) guidelines [5] with modification. For each independent experiment (6 experiments), 15-20 healthy and fertilized embryos were randomly assigned to 25 mL Pyrex glass beakers with 11 mL sterile ERM with

designated concentrations spanning environmentally relevant values of 0.01, 1, 10, 100, and 1000 μg/L PFUnDA. Fish were distributed in a randomized and staggered fashion. Fish were subjected to a cycle of 14 h light and 10 h darkness at 27 ± 1 °C. Each day of the 7-day experiment, dead embryos/larvae were removed, and an 80% water change was conducted with freshly prepared chemical dilutions. Either a Keyence All-in-One Fluorescence Microscope BZ-X710 or EVOS™ FL Auto Imaging System (Thermo Scientific, USA) were used to collect images of the embryos to document the mortality, hatch rate, and deformities. Several replicate experiments were required to obtain sufficient larvae for all toxicity assays and sub-lethal endpoints investigated in this study. Tricaine mesylate (Syncaine, Tricaine-S) was used for euthanasia at 250 mg/L buffered with equal part sodium bicarbonate to a pH between 7.0–7.5.

2.4. Acridine orange

Zebrafish larvae were exposed to 0.1% DMSO, ERM, 0.01, 1, 10, 100, or 1000 μg/L PFUnDA for 7 days (*n* = 14-16/treatment). Then, they were washed with ERM and stained with 2 μg/mL AO solution (CAS 65-61-2, Sigma-Aldrich, St. Louis, MO, USA) for 30 min at ambient temperature in the absence of light. Apoptotic cells were then visualized with an EVOSTM FL Auto Imaging System (ThermoFisher Scientific, USA) using a GFP filter at 10× magnification. Fluorescence patches of vivid green color denoted apoptotic cells. The fluorescence intensity was quantified using the histogram tool of the ImageJ software (U.S. National Institutes of Health, Bethesda, MD, USA; http://rsbweb.nih.gov/ij/).

2.5. Reactive oxygen species

Embryos were obtained for ROS assessment as outlined above in section 2.2. Embryos at 6 hours post-fertilization were washed 3 times in ERM and fertilized eggs were distributed evenly among small beakers using sterile micropipettes, each containing ~30 embryos in 15 mL of an assigned chemical concentration. The exposure concentrations were 0.01, 1, 10, 100, and 1000 μg/L PFUnDA (*n* = 3 to 5 beakers per experimental group). Following exposure, zebrafish larvae were transferred to 1.7 mL microcentrifuge tubes, flash frozen in liquid nitrogen, and homogenized in 200 μL of ice-cold PBS. Samples were then centrifuged at 12,000 g for 20 min at 4° C (LYNX6000, Thermo Scientific). For the ROS fluorescence assay, 20 μL of supernatant was transferred to a black fluorescence plate and incubated at room temperature for 5 minutes. After incubation, 8.3 μL of H2-DCFDA (1 mg/mL) and 200 μL of PBS were added. The mixture was incubated in the dark for 30 min at 37 ± 1.0 °C and then the contents were measured with an excitation at 485 nm and emission at 520 nm using a multi-detection microplate reader (New Synergy™ 4, BioTek). Total protein was determined for each sample using a BCA assay (Thermo Scientific) and ROS levels were expressed as normalized signal intensity/(μg/mL) protein.

2.6. Real-time PCR analysis

To conduct real-time PCR assays, a double batch of fish were bred as per section 2.2. This breeding event included 2 different sets of parents and yielded over 800 eggs. After the toxicity assay, a subset of fish was flash frozen in liquid nitrogen. Samples were then stored at −80 °C for ribonucleic acid (RNA) extraction and real time PCR analysis (0.1% DMSO, 1 μg/L, and 100 μg/L PFUnDA; *n* = 3 or 4 biological replicates per group). Biological replicates varied due to balancing replicates (beakers), concentrations, and larval fish numbers. Fish were treated for 7 days to PFUnDA prior to sample collection.

Real-time PCR followed our established protocols using TRIzol® Reagent (Life Technologies, Carlsbad, CA, USA) [6]. Following extraction using TRIzol® Reagent, RNA integrity was determined using the RNA 6000 nano kit and 2100 Bioanalyzer (Agilent Technologies, Santa Clara, CA, USA). Samples with RNA integrity values greater than 7 were

used for downstream analyses. Genomic DNA was removed using the TURBO DNA free™ Kit as per manufacturer's instructions (ThermoFisher Scientific). The complementary deoxyribonucleic acid (cDNA) step was conducted using 500 ng RNA and the iScript™ Select cDNA Synthesis Kit (Bio-Rad, CA, USA). Three "no reverse transcriptase (NRT)" controls were prepared in the same fashion except water was used instead of enzyme. The T100™ Thermal Cycler (BioRad, USA) was used to cycle temperatures needed to generate cDNA as per manufacturer's instruction. The CFX Connect System (BioRad, USA) was used to perform reverse transcription quantitative polymerase chain reaction (RT-qPCR) with SSo-Fast™ EvaGreen® Supermix Kit (BioRad, Hercules, CA, USA). Samples were run in duplicate and followed RT-qPCR cycling parameters described by us [7].

Primers used in this study were obtained from published literature **(Supplemental Table 1)** [7-22]. The transcripts measured in this study included *ache* (acetylcholinesterase), *atp06* (ATP synthase F0 subunit 6), *bcl2* (B-cell lymphoma 2), *bdnf* (brain-derived neurotrophic factor), *casp3* (caspase 3), *cat* (catalase), *ctgfa* (connective tissue growth factor a), *elavl3* (ELAV-like RNA binding protein 3), *gap43* (growth-associated protein 43), *gfap* (glial fibrillary acidic protein), *gmfb* (glia maturation factor beta), *ho1* (heme oxygenase 1), *keap1* (Kelch-like ECH-associated protein 1), *manf* (mesencephalic astrocyte-derived neurotrophic factor), *mbp* (myelin basic protein), *nestin* (intermediate filament protein nestin), *nkx2-2a* (NK2 homeobox 2a), *nqo1* (NAD(P)H quinone dehydrogenase 1), *nrf2* (nuclear factor erythroid 2-related factor 2), *sod1* (superoxide dismutase 1), *sod2* (superoxide dismutase 2), *sox 19b* (SRY-box transcription factor 19b), and *tubulin* (tubulin). *Rps18* (ribosomal subunit 18) and *bactin* (beta-actin) were used to normalize expression levels of all target genes using the CFX Manager (v3.1) software. Normalized expression was obtained for each target gene using CFX Manager™ software (v3.1) (baseline subtracted) and the cycle threshold (Cq) method was employed.

2.7. Visual motor response test

The Visual Motor Response (VMR) test was employed to test for differences in locomotor activity after exposure to PFUnDA. Zebrafish embryos were collected for the VMR test after multiple embryo toxicity tests. The experimental groups included ERM, 0.1% DMSO, 0.01, 1, 10, 100, and 1000 μg/L PFUnDA. Multiple replicate beakers were used for each toxicity experiment (n=5-10 in each experiment).

Larvae were exposed continuously for 7 days to designated concentrations in glass beakers containing 11 mL ERM. In each trial, ~800 zebrafish embryos at 6 hpf were randomly assigned to an experimental group of either ERM control or one concentration of 0.1-1000 μg/L PFUnDA (n = 120-200 fish derived from replicate beakers/treatment, in 3 independent experiments). An 80% daily water change was conducted daily to renew the PFUnDA. The experiments were conducted at a temperature of 27 ± 1 °C and photoperiod of 14:10 h in benchmark mini ovens.

On the seventh day in the mid-afternoon, 2 to 3 normally developed larvae were selected from each replicate beaker and individually placed into a 96-well plate (n = 16 individuals/treatment). Each well contained 200 μL of ERM. The 96-well plate was placed into DanioVision™ Observation Chamber (Noldus Information Technology, Leesburg, VA) with an infrared analog camera (25 frames/second) to track larval locomotor activity. The VMR test proceeded as per our established methods [2, 6, 23, 24]. For each trial, the 0.1% DMSO group was adjusted to become a relative measure of 1 (by dividing individual values by the mean of the group), and all other experimental groups were adjusted to be relative the DMSO control (by dividing data for each fish by the mean DMSO control value). In this manner, the three experiments could be combined into a single graph.

2.8. Statistical analysis

Statistical analysis and graphing were conducted using GraphPad v9.5.1 (La Jolla, CA, USA). Data were first assessed for normality using a Shapiro-Wilk test and ROS, gene expression data, and "distance moved" (locomotor activity) were log10 transformed to approximate a normal distribution. Survival was analyzed with a Kaplan-Meier test (log-rank Mantel-Cox test). Statistical hypothesis testing was not employed to analyze deformity and hatch data as hatch rate was observably unaltered, and deformity frequency was low (<2%) in all treatment groups. Apoptosis (AO stain), ROS [relative fluorescence units (μg/mL protein)], gene expression levels, and the VMR for larval zebrafish were analyzed using a One-Way ANOVA, followed by a Dunnett's multiple comparisons test to the control group (mean ± SD). Significance of difference was determined to be $p < 0.05$.

References

1. Westerfield, M., *The zebrafish book: a guide for the laboratory use of zebrafish (Brachydanio rerio) University of Oregon Press.* Eugene, OR, USA, 1995.
2. Huang, T., et al., *Behavioral and developmental toxicity assessment of the strobilurin fungicide fenamidone in zebrafish embryos/larvae (Danio rerio).* Ecotoxicology and Environmental Safety, 2021. **228**: p. 112966.
3. Perez-Rodriguez, V., et al., *Tebuconazole reduces basal oxidative respiration and promotes anxiolytic responses and hypoactivity in early-staged zebrafish (Danio rerio).* Comparative Biochemistry and Physiology Part C: Toxicology & Pharmacology, 2019. **217**: p. 87-97.
4. Kimmel, C.B., et al., *Stages of embryonic development of the zebrafish.* Developmental dynamics, 1995. **203**(3): p. 253-310.
5. OECD, T.N., *236: Fish embryo acute toxicity (FET) test.* OECD Guidelines for the Testing of Chemicals, Section, 2013. **2**: p. 1-22.
6. Liang, X., et al., *Biological effects of the benzotriazole ultraviolet stabilizers UV-234 and UV-320 in early-staged zebrafish (Danio rerio).* Environmental pollution, 2019. **245**: p. 272-281.
7. Wang, X.H., et al., *Paraquat affects mitochondrial bioenergetics, dopamine system expression, and locomotor activity in zebrafish (Danio rerio).* Chemosphere, 2018. **191**: p. 106-117.
8. Jin, Y., et al., *Effect of endocrine disrupting chemicals on the transcription of genes related to the innate immune system in the early developmental stage of zebrafish (Danio rerio).* Fish & shellfish immunology, 2010. **28**(5-6): p. 854-861.
9. Fang, C., et al., *6PPD induced cardiac dysfunction in zebrafish associated with mitochondrial damage and inhibition of autophagy processes.* Journal of Hazardous Materials, 2024. **471**: p. 134357.
10. Maffioli, E., et al., *Brain proteome and behavioural analysis in wild type, BDNF+/− and BDNF−/− adult zebrafish (Danio rerio) exposed to two different temperatures.* International Journal of Molecular Sciences, 2022. **23**(10): p. 5606.
11. Sarkar, S., et al., *Low dose of arsenic trioxide triggers oxidative stress in zebrafish brain: expression of antioxidant genes.* Ecotoxicology and environmental safety, 2014. **107**: p. 1-8.
12. Mukherjee, D., et al., *Ccn2a is an injury-induced matricellular factor that promotes cardiac regeneration in zebrafish.* Development, 2021. **148**(2): p. dev193219.
13. Yang, Q., et al., *Developmental neurotoxicity of difenoconazole in zebrafish embryos.* Toxics, 2023. **11**(4): p. 353.
14. Dong, M., et al., *3-bromocarbazole-induced developmental neurotoxicity and effect mechanisms in zebrafish.* Acs Es&T Water, 2023. **3**(8): p. 2471-2480.
15. Li, H.-Y., et al., *Glia maturation factor-β in hepatocytes enhances liver regeneration and mitigates steatosis and ballooning in zebrafish.* American Journal of Physiology-Gastrointestinal and Liver Physiology, 2025.
16. Li, X., et al., *SOX19b regulates the premature neuronal differentiation of neural stem cells through EZH2-mediated histone methylation in neural tube development of zebrafish.* Stem cell research & therapy, 2019. **10**: p. 1-14.
17. Wang, J., et al., *Developmental toxicity of Zishen Guchong Pill on the early life stages of Zebrafish.* Phytomedicine Plus, 2021. **1**(4): p. 100088.
18. Zhao, X., et al., *Quercetin mitigates ethanol-induced hepatic steatosis in zebrafish via P2X7R-mediated PI3K/Keap1/Nrf2 signaling pathway.* Journal of ethnopharmacology, 2021. **268**: p. 113569.
19. Jiang, F., et al., *Tris (2-butoxyethyl) phosphate affects motor behavior and axonal growth in zebrafish (Danio rerio) larvae.* Aquatic Toxicology, 2018. **198**: p. 215-223.
20. Fan, C.-Y., et al., *Gene expression changes in developing zebrafish as potential markers for rapid developmental neurotoxicity screening.* Neurotoxicology and teratology, 2010. **32**(1): p. 91-98.

21. McCurley, A.T. and G.V. Callard, *Characterization of housekeeping genes in zebrafish: male-female differences and effects of tissue type, developmental stage and chemical treatment.* BMC molecular biology, 2008. **9**: p. 1-12.
22. Wu, Q., et al., *Microcystin-LR exposure induces developmental neurotoxicity in zebrafish embryo.* Environmental Pollution, 2016. **213**: p. 793-800.
23. Huang, T., et al., *Exposure to acetochlor impairs swim bladder formation, induces heat shock protein expression, and promotes locomotor activity in zebrafish (Danio rerio) larvae.* Ecotoxicology and Environmental Safety, 2021. **228**: p. 112978.
24. Huang, T., et al., *Behavioral and developmental toxicity assessment of the strobilurin fungicide fenamidone in zebrafish embryos/larvae (Danio rerio).* Ecotoxicology and Environmental Safety, 2021. **228**: p. 112966.

Disclaimer/Publisher's Note: The statements, opinions and data contained in all publications are solely those of the individual author(s) and contributor(s) and not of MDPI and/or the editor(s). MDPI and/or the editor(s) disclaim responsibility for any injury to people or property resulting from any ideas, methods, instructions or products referred to in the content.
