# Supplementary material for: Neurotoxicity Assessment of Perfluoroundecanoic Acid (PFUnDA) in Developing Zebrafish (Danio rerio)
Source: Toxics. 2025 Nov 22;13(12):1012. doi: 10.3390/toxics13121012 (PMC12737132; doi:10.3390/toxics13121012)
Supplement: Supplementary file 1 [file toxics-13-01012-s001.zip › Suppl. Table 1.pdf]

Supplemental Tables

Article

Neurotoxicity Assessment of Perfluoroundecanoic Acid (PFUnDA) in Developing Zebrafish (*Danio rerio*)

Lev Avidan [1,†], Cole D. English [1,†], Emma Ivantsova [1], Amany Sultan [1,2] and Christopher J. Martyniuk [1,3,*]

[1] Center for Environmental and Human Toxicology, Department of Physiological Sciences, College of Veterinary Medicine, University of Florida, Gainesville, FL 32611, USA; lavidan@ufl.edu (L.A.); coleenglish@ufl.edu (C.D.E.); eivantsova@ufl.edu (E.I.); amanysultan2025@gmail.com (A.S.)
[2] Animal Health Research Institute, Agriculture Research Centre, Giza 12619, Egypt
[3] UF Genetics Institute, Interdisciplinary Program in Biomedical Sciences Neuroscience, Gainesville, FL 32611, USA
* Correspondence: cmartyn@ufl.edu; ORCID: 0000-0003-0921-4796
† These authors contributed equally to this work.

Supplemental Table S1

Primers used for real-time PCR analysis.

Gene name	Gene Symbol	Forward (5' to 3')	Reverse (3' to 5')	Reference
Acetylcholinesterase	*ache*	GCTAATGAGCAAAAGCATGTGGGC	TATCTGTGATGTTAAGCAGACGAGGCA	NM_131846.2
ATP synthase F0 subunit 6	*atp06*	TTATCCTCGTTGCCATACTTC	AGTTGGTTTGTGAATCGTCC	Jin et al., 2010
Beta-actin	*bactin*	CGAGCAGGAGATGGGAACC	CAACGGAAACGCTCATTGC	Wang et al. 2018
BCL2 Apoptosis Regulator	*bcl2*	AGGAAAATGGAGGTTGGGATG	TGTTAGGTATGAAAACGGGTGGA	Fang et al., 2024
Brain-derived neurotrophic factor	*bdnf*	ATAGTAACGAACAGGATGG	GCTCAGTCATGGGAGTCC	NM_001308648.1<br>Maffioli et al., 2022
Catalase	*cat*	CTCCTGATGTGGCCCGATAC	TCAGATGCCCGGCCATATTC	Sarkar et al., 2014
Caspase 3, apoptosis-related cysteine peptidase	*casp3*	CCGCTGCCCATCACTA	ATCCTTTCACGACCATCT	Fang et al., 2024
Connective tissue growth factor a	*ctgfa*	GGTGTACCGCAGTGGAGAGT	CTACAGCACCGTCCAGACAC	Mukherjee et al., 2021
ELAV Like RNA Binding Protein 3	*elavl3*	AGACAAGATCACAGGCCAGAGCTT	TGGTCTGCAGTTTGAGACCGTTGA	NM_131449.1<br>Yang et al. 2023
Growth Associated Protein 43	*gap43*	TTAACGGAGGACCAGTGCAA	GTCCTGATCTCCAGCACACG	NM_131341.1<br>Dong et al., 2023
Glial Fibrillary Acidic Protein	*gfap*	GGATGCAGCCAATCGTAAT	TTCCAGGTCACAGGTCAG	NM_131373.2<br>Dong et al., 2023

Glia maturation factor beta	*gmfb*	TCCTGTTGGATGCAAACCTGAGCA	GCGAAAGAATCCTAGTTTCTCCCGG	Li et al., 2025
Heme oxygenase1	*ho1*	AAGCAAAGCGGCAGAGAAC	TGGAGCAGTCAGATGAAGTGT	Wang et al., 2021
Kelch-like ECH-associated protein 1	*keap1*	CCAACGGCATAGAGGTAGTTAT	CCTGTATGTGGTAGGAGGGTT	Zhao et al., 2021
Mesencephalic Astrocyte-Derived Neurotrophic Factor	*manf*	AGATGGAGAGTGTGAAGTCTGTGTG	CAATTGAGTCGCTGTCAAAACTTG	NM_001076629<br>Yang et al., 2023
Myelin Basic Protein	*mbp*	AATCAGCAGGTTCTTCGGAGGAGA	AAGAAATGCACGACAGGGTTGACG	BC115202.1<br>Yang et al., 2023
Nestin	*nestin*	ATGCTGGAGAAACATGCCATGCAG	AGGGTGTTTACTTGGGCCTGAAGA	XM_001919887<br>Jiang et al., 2018
Nuclear factor erythroid 2 related factor 2	*nrf2*	AAGCAGACGGAGGAGGAG	GGAGGTGTTCAGGCAAGG	Wang et al., 2021
NAD(P)H dehydrogenase, quinone 1	*nqo1*	CGCGAGATGTTGCAGTTCAG	AAGTGCTCGGGATTCTGCAA	Zhao et al., 2021
NK2 homeobox 2a	*nkx2-2a*	CGGCAAACCTTGCCATACGCTAAA	GCGCGTTATATTGCATGTGCTGGA	Fan et al., 2010
Ribosomal protein S18	*rps18*	TCGCTAGTTGGCATCGTTTATG	CGGAGGTTCGAAGACGATCA	McCurley and Callard, 2008

Superoxide dismutase 1	*sod1 (Cu/Zn SOD)*	CAACACAAACGGCTGCATCA	TTTGCAACACCACTGGCATC	Sarkar et al., 2014
Superoxide dismutase 2	*sod2 (Mn SOD)*	AGCGTGACTTTGGCTCATTT	ATGAGACCTGTGGTCCCTTG	Sarkar et al., 2014
SRY-box transcription factor 19b	*sox 19b*	AAATATCCTCTTGCAGCGGG	CTGTTCATGTAGGGCTGTGC	Li et al., 2019
Tubulin	*tubulin*	AATCACCAATGCTTGCTTCGAGCC	TTCACGTCTTTGGGTACCACGTCA	NM_194388<br>Wu et al., 2016

References

1. Dong, M., Wang, J., Liu, Y., He, Q., Sun, H., Xu, Z., ... & Gao, P. (2023). 3-Bromocarbazole-Induced Developmental Neurotoxicity and Effect Mechanisms in Zebrafish. *ACS ES&T Water*.
2. Fan, C. Y., Cowden, J., Simmons, S. O., Padilla, S., & Ramabhadran, R. (2010). Gene expression changes in developing zebrafish as potential markers for rapid developmental neurotoxicity screening. *Neurotoxicology and Teratology*, *32*(1), 91–98. https://doi.org/10.1016/j.ntt.2009.04.065
3. Fang, C., Di, S., Yu, Y., Qi, P., Wang, X., & Jin, Y. (2024). 6PPD induced cardiac dysfunction in zebrafish associated with mitochondrial damage and inhibition of autophagy processes. *Journal of hazardous materials*, *471*, 134357. https://doi.org/10.1016/j.jhazmat.2024.134357
4. Jin, Y.; Chen, R.; Liu, W.; Fu, Z. Effect of endocrine disrupting chemicals on the transcription of genes related to the innate immune system in the early developmental stage of zebrafish (Danio rerio). Fish Shellfish Immunol. 2010, 28, 854–861.
5. Jiang, F., Liu, J., Zeng, X., Yu, L., Liu, C. and Wang, J., 2018. Tris (2-butoxyethyl) phosphate affects motor behavior and axonal growth in zebrafish (Danio rerio) larvae. *Aquatic Toxicology*, *198*, pp.215-223.
6. Li, X., Zhou, W., Liu, X., Zhang, Y., & Hao, A. (2019). SOX19b regulates the premature neuronal differentiation of neural stem cells through EZH2-mediated histone methylation in neural tube development of zebrafish. *Stem Cell Research & Therapy*, *10*(1), 389. https://doi.org/10.1186/s13287-019-1495-3
7. Li, H.-Y., Zeng, W.-L., Ye, Y.-W., Chen, X., Zhang, M.-M., Chen, Y.-S., Liu, C.-T., Zhong, Z.-Q., Li, J., & Wang, Y. (2025). *Glia maturation factor-β in hepatocytes enhances liver regeneration and mitigates steatosis and ballooning in zebrafish*. *American Journal of Physiology – Gastrointestinal and Liver Physiology*. Advance online publication. https://doi.org/10.1152/ajpgi.00407.2024
8. Maffioli, E., Angiulli, E., Nonnis, S., Grassi Scalvini, F., Negri, A., Tedeschi, G., Arisi, I., Frabetti, F., D'Aniello, S., Alleva, E., Cioni, C., & Toni, M. (2022). Brain Proteome and Behavioural Analysis in Wild Type, BDNF+/- and BDNF-/- Adult Zebrafish (Danio rerio) Exposed to Two Different Temperatures. International journal of molecular sciences, 23(10), 5606. https://doi.org/10.3390/ijms23105606
9. McCurley AT, Callard GV. Characterization of housekeeping genes in zebrafish: male-female differences and effects of tissue type, developmental stage and chemical treatment. BMC Mol Biol. 2008 Nov 12;9:102. doi: 10.1186/1471-2199-9-102. PMID: 19014500; PMCID: PMC2588455.

10. Mukherjee, D., Wagh, G., Mokalled, M. H., Kontarakis, Z., Dickson, A. L., Rayrikar, A., Günther, S., Poss, K. D., Stainier, D. Y. R., & Patra, C. (2021). Ccn2a is an injury-induced matricellular factor that promotes cardiac regeneration in zebrafish. Development (Cambridge, England), 148(2), dev193219. https://doi.org/10.1242/dev.193219
11. Sarkar, S., Mukherjee, S., Chattopadhyay, A. and Bhattacharya, S., 2014. Low dose of arsenic trioxide triggers oxidative stress in zebrafish brain: expression of antioxidant genes. Ecotoxicology and environmental safety, 107, pp.1-8.
12. Wang, X.H., Souders 2nd, C.L., Zhao, Y.H., Martyniuk, C.J. 2018. Paraquat affects mitochondrial bioenergetics, dopamine system expression, and locomotor activity in zebrafish (Danio rerio). Chemosphere. 191, 106-117.
13. Wang, J., Mo, C., Tu, P., Ning, N., Liu, X., Lin, S., ... & He, Q. (2021). Developmental toxicity of Zishen Guchong Pill on the early life stages of Zebrafish. *Phytomedicine Plus*, *1*(4), 100088.
14. Wu, Q., Yan, W., Liu, C., Li, L., Yu, L., Zhao, S. and Li, G., 2016. Microcystin-LR exposure induces developmental neurotoxicity in zebrafish embryo. Environmental Pollution, 213, pp.793-800.
15. Yang Q, Deng P, Xing D, Liu H, Shi F, Hu L, Zou X, Nie H, Zuo J, Zhuang Z, Pan M, Chen J, Li G. Developmental Neurotoxicity of Difenoconazole in Zebrafish Embryos. Toxics. 2023 Apr 8;11(4):353. doi: 10.3390/toxics11040353. PMID: 37112580; PMCID: PMC10142703.
16. Zhao, X., Gong, L., Wang, C., Liu, M., Hu, N., Dai, X., Peng, C., & Li, Y. (2021). Quercetin mitigates ethanol-induced hepatic steatosis in zebrafish via P2X7R-mediated PI3K/Keap1/Nrf2 signaling pathway. *Journal of ethnopharmacology*, *268*, 113569.
